# Policies for Infection Prevention and Control in Home Health Care, 2019 vs 2023

**DOI:** 10.1001/jamanetworkopen.2025.12450

**Published:** 2025-05-27

**Authors:** Jingjing Shang, Ji Won Lee, Andrew W. Dick, Ashley M. Chastain, U. Gayani E. Perera, Laurent G. Glance, Patricia W. Stone

**Affiliations:** 1 Center for Health Policy, Columbia University School of Nursing, New York, New York; 2 The RAND Corporation, RAND Health, Boston, Massachusetts; 3 Department of Anesthesiology and Perioperative Medicine, University of Rochester School of Medicine, Rochester, New York

## Abstract

This cross-sectional study examines how policies for infection prevention and control have evolved among US home health care agencies after the COVID-19 pandemic.

## Introduction

Home health care (HHC) is vital to the US health care system, delivering skilled care to patients aged 65 years or older with acute illness. Nearly 3 million Medicare beneficiaries received care from more than 12 000 Medicare-certified HHC agencies, costing approximately $16 billion.^1^ Home health care spending is projected to outpace other sectors,^2^ driven by an aging population and a preference for in-home care, especially after COVID-19. Despite benefits, HHC patients face infection risk due to chronic conditions, wounds, medical devices, and a less controlled home environment.^3^ Infection prevention and control (IPC) is a national patient safety goal, yet a prepandemic survey found wide IPC policy variation,^4^ with deficiencies worsening during the pandemic because of personal protection equipment shortages and insufficient IPC training.^5^ We examined how HHC IPC policies evolved after the pandemic and hypothesized that IPC policies improved.

## Methods

This cross-sectional study analyzed data from nationally representative surveys of Medicare-certified HHC agencies in 2019 and 2023. The Columbia University Medical Center Institutional Review Board determined the project not to constitute human participants research; therefore, informed consent was not required under the Common Rule. The study followed the STROBE reporting guideline for cross-sectional studies.

Paper and electronic surveys covered IPC infrastructure (eg, personnel, training) and general and recommended policies across 4 key domains: antibiotic stewardship (6 items), urinary tract infection (6 items), intravenous and central catheters (5 items), and pneumonia (4 items). A composite intensity score was calculated for each domain by summing items and converting them into a binary variable of 1 (high policy intensity, ie, the 2 highest scores) and 0 (low policy intensity). Probability weights accounting for sample design and nonresponse were applied. Multivariable logistic regression models estimated associations between survey year and IPC policy intensity, adjusting for agency characteristics. Data were analyzed using Stata, version 18.0 (StataCorp LLC). *P* < .05 was considered significant.

## Results

A total of 1052 surveys were completed (536 in 2019, 474 in 2023), with 171 agencies completing both. Response rates (36% in 2019, 32% in 2023) were strong for HHC, a hard-to-reach group. Weighting addressed response bias, yielding a nationally representative sample (15% rural, 81% for-profit, and 7% hospital-affiliated agencies).

Among IPC personnel, fewer lacked IPC training in 2023 vs 2019 (14.0% vs 34.1%; *P* < .001), and hospital-provided training increased (16.8% vs 1.3%; *P* < .001) (Table). However, IPC training for all staff declined in 2023, with fewer agencies offering it during new employee orientation (52.1% vs 61.0%; *P* = .02) and virtual replacing in-person sessions. Committees reviewed IPC policies less often in 2023 (64.5% vs 70.5%; *P* < .001). More agencies cited field staff coverage as the most challenging IPC issue in 2023 (40.5% vs 21.4%; *P* < .001).

**Table.** IPC Personnel, Activities, and Training Among HHC Agencies

Variable	HHC agencies, No. (weighted %)		
	2019 (n = 536)	2023 (n = 474)	*P* value
**Personnel in charge of IPC activities**			
Hours spent on IPC for person in charge of infection prevention, mean (SD)	8.4 (30.3)	10.5 (11.6)	.27
Highest level of education			
Associate’s degree	112 (22.5)	94 (20.1)	.70
Bachelor’s degree	276 (55.3)	253 (53.9)	
Master’s degree	76 (15.4)	83 (17.7)	
Doctorate	5 (1.0)	11 (2.3)	
Other^a^	8 (1.6)	8 (1.7)	
Do not know	21 (4.2)	21 (4.4)	
Specific infection control training			
Certified in infection control	34 (6.8)	34 (7.3)	.80
Agency training through an external consultant	181 (36.4)	180 (38.4)	.60
Training through the hospital	6 (1.3)	79 (16.8)	<.001
External training through professional society, department of health, or other organization	141 (28.3)	165 (35.3)	.06
Other^b^	20 (4.0)	40 (8.6)	.02
No training	170 (34.1)	66 (14.0)	<.001
**IPC activities**			
IPC assessment			
Patient risk factors for infection	482 (90.7)	434 (91.7)	.65
Cohabiting individuals	276 (51.9)	249 (52.6)	.86
Noncompliance with IPC procedures	395 (74.3)	305 (64.5)	<.001
Most time-consuming IPC activities^c^			
Staff education	457 (85.7)	398 (84.2)	.59
Monitoring adherence to IPC practices	384 (72.1)	322 (68.0)	.24
Data collection and reporting	435 (81.7)	338 (71.5)	<.001
Patient vaccination monitoring	79 (14.8)	73 (15.4)	.82
Employee vaccination monitoring	86 (16.1)	108 (22.9)	.03
Most challenging IPC activity^d^			
Adherence to and monitoring hand hygiene and standard precautions	86 (16.2)	93 (7.1)	<.001
Adherence to bag technique	122 (23.1)	73 (15.5)	.02
Collecting and reporting infection data	202 (38.2)	92 (19.5)	<.001
Treating patients with multidrug-resistant organisms (ie, *Clostridioides difficile*)	75 (14.2)	11 (2.3)	<.001
Adequate field staffing coverage	114 (21.4)	192 (40.5)	<.001
**IPC training**			
IPC training format			
Face-to-face training	486 (91.1)	401 (84.7)	.01
Computer-based training tools (ie, online modules, DVDs)	348 (65.3)	355 (75.0)	.01
Shadowing in the field	302 (56.6)	234 (49.6)	.07
Knowledge assessment	303 (56.8)	218 (46.2)	.01
No IPC training	2 (0.3)	0	.39
IPC training frequency			
Annually	342 (64.1)	330 (69.7)	.14
Quarterly	127 (23.8)	106 (22.3)	.65
Monthly	73 (13.6)	58 (12.2)	.59
At new employee orientation	325 (61.0)	247 (52.1)	.02
When an infection control issue or outbreak arises	252 (47.4)	201 (42.4)	.20
**IPC committee**			
Committee within HHC agency that reviews IPC-related activities and issues			
Yes	372 (70.5)	306 (64.50)	<.001
No, but there are plans	51 (9.6)	24 (5.1)	
No, there are no future plans	82 (15.5)	125 (26.4)	
Other^e^	10 (1.9)	12 (2.6)	
Do not know	13 (2.5)	7 (1.4)	
Frequency of committee meeting			
Annually	23 (6.4)	55 (13.0)	<.001
Quarterly	241 (68.2)	255 (59.9)	
Monthly	80 (22.7)	78 (18.3)	
No regular meetings	2 (0.52)	10 (2.43)	
Other^f^	4 (1.0)	9 (2.0)	
Do not know	4 (1.1)	18 (4.3)	
**HHC agencies with high infection–specific policy intensity^g^**			
Antibiotic stewardship	120 (22.4)	41 (10.6)	<.001
Urinary tract infection	229 (43.2)	201 (42.9)	.93
Intravenous and central catheter infection	315 (64.8)	219 (51.0)	<.001
Pneumonia	305 (57.0)	226 (47.7)	.02

Abbreviations: DVD, digital video disk; HHC, home health care; IPC, infection prevention and control.

^a^ Other education included emergency medical care/licensed practical nurse certification and diploma of nursing.

^b^ Other specific infection control training included, in-services, self-taught, and other modes.

^c^ Respondents listed 3 time-consuming activities.

^d^ Respondents selected 1 challenging IPC activity.

^e^ Other committee IPC-related activities and issues included hospital-based committee that agency was a member of.

^f^ Other frequency of committee meetings included as-needed, sporadically, and other options.

^g^ A composite intensity score was calculated by summing items for each domain and converting it into a binary variable: 1 (high policy intensity, top 2 scores) or 0 (low policy intensity, the rest of the scores). Thresholds for antibiotic stewardship were the following: urinary tract infection (6 items), 1 (5-6 policies) and 0 (0-4 policies); intravenous and central catheter infection (5 items), 1 (4-5 policies) and 0 (0-3 policies); and pneumonia (4 items), 1 (3-4 policies) and 0 (0-2 policies).

Declines in policy intensity occurred across 3 of the 4 domains (Figure). Agencies with high antibiotic stewardship policy intensity fell from an adjusted 23.4% (2019) to 11.8% (2023). Similarly, high policy intensity for intravenous and central catheter infections (from 68.5% to 56.1%) and pneumonia (from 65.9% to 52.4%) also decreased (*P* < .001). A sensitivity analysis using longitudinal data of 171 agencies revealed similar results.

**Figure.**
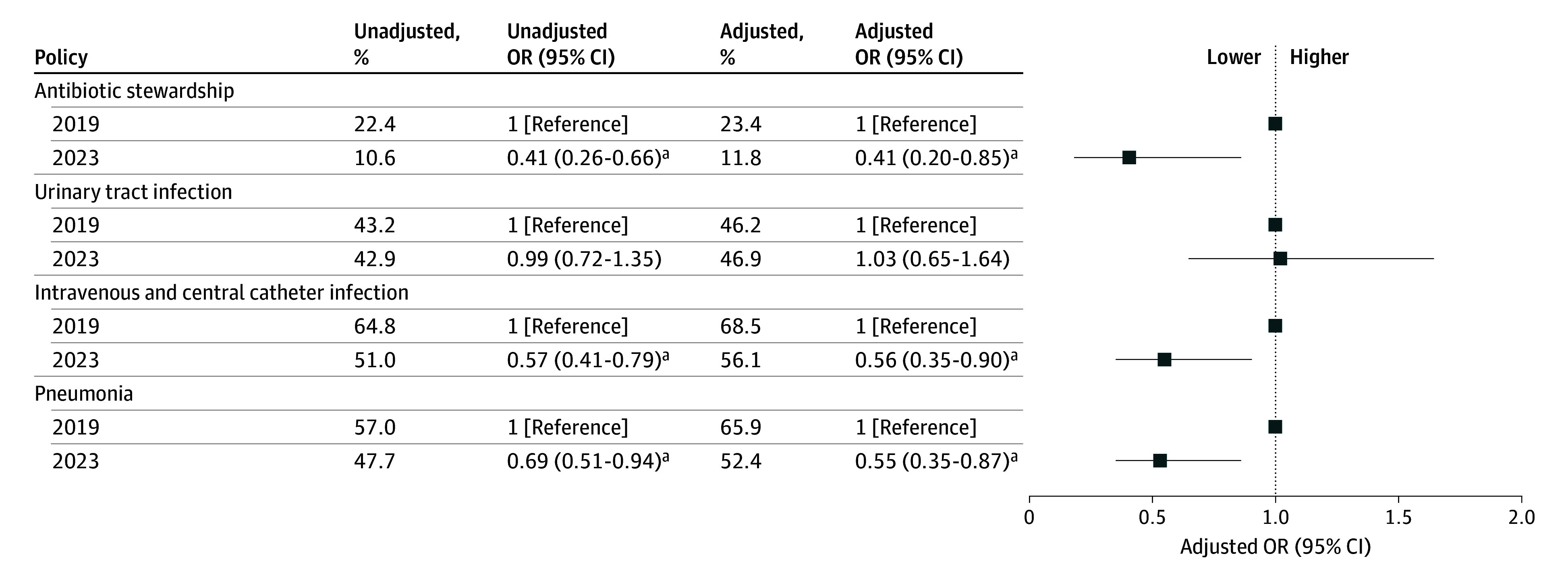
Association Between High Policy Intensities of Infection Prevention and Control–Related Policies, 2023 vs 2019 Odds ratios (ORs) were calculated by estimating univariable and multivariable logistic regressions (adjusting for home health care agency characteristics). Estimated models were derived from the corresponding models. ^a^Significant difference.

## Discussion

This cross-sectional study revealed serious declines and persistent gaps in IPC in HHC agencies, with only minor improvements, opposite to our hypothesis. The IPC training decline for all staff may reflect shifts in focus to vaccination surveillance. Significant declines in antibiotic stewardship, intravenous and central catheter infection, and pneumonia prevention policies raise concerns about increased infection risks for vulnerable HHC patients. Despite growing recognition of IPC importance during the pandemic, key policies have weakened since, perhaps due to workforce shortages and persistent resource strain,^6^ hindering effective implementation. Addressing these challenges requires coordinated and systematic actions. This research was limited by subjective IPC measures; thus, future research should use objective measures to address bias. Investing in workforce capacity, resources, and training is essential for safe and high-quality HHC.

## Notes

Supplement.
Data Sharing Statement
