# Supplementary material for: Policies for Infection Prevention and Control in Home Health Care, 2019 vs 2023
Source: JAMA Netw Open. 2025 May 27;8(5):e2512450. doi: 10.1001/jamanetworkopen.2025.12450 (PMC12117440; doi:10.1001/jamanetworkopen.2025.12450)
Supplement: Supplement. — Data Sharing Statement [file jamanetwopen-e2512450-s001.pdf]

Data Sharing Statement

Shang. Policies for Infection Prevention and Control in Home Health Care, 2019 vs 2023. *JAMA Netw Open*. Published May 27, 2025. doi:10.1001/jamanetworkopen.2025.12450

Data
Data available: No

Additional Information
Explanation for why data not available: No, the survey data will not be shared as per the consent agreement signed by participants, ensuring confidentiality and adherence to ethical guidelines
